# A Tele‐Coaching Pilot Study: An Innovative Approach to Enhance Motor Skills in Adolescents With Down Syndrome

**DOI:** 10.1111/jar.70036

**Published:** 2025-03-07

**Authors:** Matteo Giuriato, Alessandro Gatti, Vittoria Carnevale Pellino, Alice Bianchi, Sara Zanelli, Agnese Pirazzi, Caterina Cavallo, Antonia Quatrale, Alessandra Anna Gazzarri, Matteo Vandoni, Gianvincenzo Zuccotti, Valeria Calcaterra

**Affiliations:** ^1^ Laboratory of Adapted Motor Activity (LAMA), Department of Public Health, Experimental Medicine and Forensic Science University of Pavia Pavia Italy; ^2^ Pediatric Department Buzzi Children's Hospital Milan Italy; ^3^ Department of Sport and Exercise Science LUNEX International University of Health, Exercise and Sports Differdange Luxembourg; ^4^ Associazione Vivi Down Onlus Milan Italy; ^5^ Department of Biomedical and Clinical Science University of Milan Milan Italy; ^6^ Pediatric and Adolescent Unit, Department of Internal Medicine University of Pavia Pavia Italy

**Keywords:** adolescents, down syndrome, motor competencies, online training, physical fitness

## Abstract

**Background:**

Limited knowledge exists regarding the effectiveness of training programmes for individuals with Down syndrome, particularly innovative approaches like tele‐coaching. Our pilot study aimed to improve strength and balance using tele‐coaching sessions in children with Down syndrome.

**Materials and Methods:**

We enrolled 18 children and adolescents (aged 9–17 years) with Down syndrome. The intervention consisted of a training programme based on games and was conducted remotely through an online platform (e‐gym) 3 days per week (15 weeks). Participants engaged in playful activities targeting limb strength and balance.

**Results:**

We found an improvement in systolic blood pressure (*p* = 0.04) and balance (*p* = 0.002). Our analysis showed a non‐significant decrease in adiposity parameters, including weight, BMI, BMI *z*‐score, WC and WC/H.

**Conclusions:**

Our findings contribute to evidence supporting online exercise interventions for individuals with Down syndrome. Integrating these interventions into community support programmes could enhance access to tailored services.

**Summary**

- Main findings.
  ○ Tele‐coaching interventions significantly improved blood pressure and balance among adolescents with Down syndrome. While there was a trend towards decreased adiposity parameters, no significant differences were observed in cognitive domains.
  ○ This pilot study highlights the potential of tele‐coaching to address physical health challenges in individuals with Down syndrome, offering a promising avenue for promoting cardiovascular health and motor skills development.
- Impact of our findings on people with Down syndrome.
  ○ Our findings suggest that tele‐coaching interventions can be effective in improving cardiovascular health and balance, addressing the specific health needs of individuals with Down syndrome.
  ○ By providing remote access to structured exercise programmes, tele‐coaching offers a convenient and accessible option for individuals with Down syndrome to engage in physical activity, promoting overall health and well‐being.

## 1 Introduction

Exercise and structured physical activity play an important role in the development and maintenance of physical health in individuals with Down syndrome (Bull 2020; Pitetti et al. 2013). However, access to adequate and adapted training programmes for this population remains limited (Maïano et al. 2019; Pitetti et al. 2013; Saquetto et al. 2018).

Down syndrome presents several challenges, notably cognitive and motor deficits that can impede participation and achievement in physical activities (Vandoni et al. 2023). These challenges encompass aspects such as physical activity engagement, motor skill development, executive function and anthropometric characteristics, including excess adiposity.

Previous research has highlighted the beneficial potential of exercise in the population with Down syndrome, improving aspects such as motor coordination, muscle strength and cardiorespiratory capacity (Gandy et al. 2020; Paul et al. 2019).

Particularly, these studies may include various activities such as hippotherapy, aquatic therapy, resistance and balance training (Azab et al. 2022; Naczk et al. 2021; Saquetto et al. 2018).

Furthermore, it is important to focus on developing fine motor skills and balance, which are particularly difficult for children with Down syndrome due to their low muscle tone and/or hypermobility in the hands, wrists or elbows (Alesi et al. 2022; Malak et al. 2015).

Gross and fine motor skills and conditional activity such as strength and balance are critical to the everyday lives of children and adolescents with Down syndrome because they directly affect their ability to perform daily tasks and participate in social activities (Alesi et al. 2022). Particularly, these skills allow subjects with Down syndrome to perform daily activities such as dressing, eating, washing and using the bathroom independently (Baumer and Davidson 2014). Without good motor control, people with Down syndrome may encounter difficulty in these activities, necessitating support from adults. Participation in exercise may lead to positive changes in cognitive functions in individuals with Down syndrome (Nocera et al. 2018), also improving social interaction. Additionally, as reported in studies with adults, maintaining good standing balance is crucial for sustaining an appropriate walking speed, which can help reduce cardiovascular risk (Montero‐Odasso et al. 2022; Yamamoto et al. 2014). Social integration can be promoted through participation in sports activities with peers at a young age, up to the entry into the world of work in adulthood.

However, there are several barriers to exercise for children and adolescents with Down syndrome, given their limited personal autonomy. In fact, Barr and Shields (2011) found that parents of youth with Down syndrome complain about a lack of mainstream programmes willing to accommodate their son and daughter, attributing this to a lack of trained staff, time constraints and poor education on the subject. Some parents feel that the lack of publicised events affects their active search for programmes, as they do not feel welcomed, expressing frustration with preconceived stereotypes and the use of the term ‘disability’, believing that this automatically generates negative and exclusive attitudes. These factors hinder their children's participation in formal activities (MacDonald et al. 2016; Van Herwegen et al. 2018).

Considering these, one of the methods of growing interest in the world of adapted physical activity is tele‐coaching (Ousley et al. 2023; Platini et al. 2022; Wingo et al. 2020). In fact, this type of online coaching allows you to connect from home without travelling.

However, there is still limited knowledge about the effectiveness of training programmes specifically designed for the Down syndrome population, especially when it comes to using innovative approaches such as the tele‐coaching programme.

Considering this, the purpose of this preliminary and pilot study intervention includes training sessions structured in such a way as to stimulate strength and balance in the upper and lower limbs, using tele‐coaching. The effects of exercise on cognitive performance, balance impairment and cardiovascular health were also considered. The remote approach can offer a unique opportunity to engage adolescents with Down syndrome in a familiar and comfortable environment, reducing the barriers to entry associated with traditional gym sessions.

The hypothesis of this preliminary and pilot study is that a structured tele‐coaching intervention aimed at improving strength and balance and evaluating the influence of physical activity on cognitive performance and cardiovascular health in adolescents with Down syndrome. The utilisation of tele‐coaching is intended to provide an innovative opportunity to engage adolescents with Down syndrome in a familiar and comfortable context, thereby overcoming the barriers commonly associated with traditional gymnasium training sessions.

## 2 Methods

### 2.1 Participants

This study represents a pilot study, and the results should be considered preliminary to a longer study. We enrolled 18 children and adolescents (aged 9–17 years) with Down syndrome referred to the outpatient clinic of Buzzi Children's Hospital in Milan, Italy. Participants were excluded from the study if they met any of the following criteria: (1) severe cognitive difficulties that impair the comprehension of instructions during testing and activities, (2) atlantoaxial instability, (3) cardiovascular diseases (CVDs) (congenital heart malformations, etc.), (4) metabolic disorders (diabetes, thyroid disease, etc.), (5) toxic habits (smoking or alcohol), (6) dietary supplements, (7) participation in a training programme in the 6 months before entering into the study and (8) not having completed at least 90% of the training sessions. In all participants' auxological assessment was performed. In previous studies, it has been demonstrated that the exclusion of participants with specific medical conditions may be necessary to maintain methodological consistency, although this may limit the generalisability of the results (Salmasi et al. 2022).

All subjects were included in one group that completed all protocols through tele‐coaching.

The study received approval from the Institutional Review Board (protocol number: 2020/ST/298; 2023/EM/099, CE Area 1 Milan, Italy) and was conducted in accordance with the Declaration of Helsinki, as revised in 2013. Upon receiving information regarding the study's nature, written informed consent was obtained from the parents/authorised caregivers of the participating children.

### 2.2 Auxological Evaluation

Auxological measurements, comprising height (H), weight, body mass index (BMI), waist circumference (WC), WC/height ratio, pubertal stage and blood pressure (BP), were conducted (Calcaterra et al. 2022; Calcaterra, Gazzarri, et al. 2023; Calcaterra, Schneider, et al. 2023). Height was assessed with the patient standing barefoot utilising a Harpenden stadiometer. Weight was measured with subjects in underwear and standing upright on a platform scale. WC was measured in the horizontal plane midway between the lowest rib and the iliac crest using a flexible steel tape. BMI was computed as weight divided by height squared and standardised into BMI *z*‐scores utilising WHO reference values (de Onis 2007). The pubertal staging was evaluated using the Marshall and Tanner classification (Marshall and Tanner 1969, 1970). BP was taken twice on each arm using a mercury sphygmomanometer after the patient had been comfortably seated for 5 min. The second reading from the right arm was used for analysis.

### 2.3 Intervention Programme

The intervention consisted of a training programme based on games and conducted remotely via an online platform, called e‐gym. The training protocol consisted of 60‐min sessions for 3 days a week for 15 weeks.

During the programme, participants were engaged in playful activities aimed at stimulating strength in the upper and lower limbs and balance. These activities were structured in virtual stations, each corresponding to a specific game, such as movement games for the arms, balance games, and so forth.

Each training session is game‐based and consists of three parts: (i) 10 min of warm‐up based, (ii) 20 min of exercises and games focused on developing strength, motor skills and balance and (iii) 10 min of cool‐down based on muscle stretching exercises.

Every 10 min, subjects could drink. The exercises were proposed as playful activities and did not require any equipment. In addition, training sessions were conducted in small groups, with a supervision ratio of one monitor per three participants, to ensure that the exercises were performed correctly and with the appropriate intensity.

Table 1 reports an example of the structure and type of exercises performed during the training sessions.

**TABLE 1:** Example of a typical training session.

	Warm‐up (10 min)	Muscular strength (10 min)	Coordination and gross motor skill training (10 min)	Cool‐down (10 min)
Type of exercises	Mobility exercises (e.g., head, lower and upper body mobility routine)	Strength circuit for children (e.g., semi‐squat, push‐up, glute bridge, triceps dip. All exercises were performed free weight)	Animal walks, imitation games, storytelling games	Yoga for children (e.g., child pose, reverse warrior, cobra pose, seated forward fold, corpse pose)

It is important to note that before the start of the training programme, the participants included in the intervention group participated in a pre‐workout session of introduction to the games and performed a test to determine the maximum number of repetitions for each exercise. Moreover, the coaches who followed the participants remotely always ensured that everyone could understand the instructions and adapted the exercises' level when needed.

All the sessions were conducted and supervised by four coaches who graduated in sports sciences.

### 2.4 Gross Motor Coordination

GMC was assessed using a novel and condensed version of the KTK battery (Kiphard and Schilling 2007) that is a valid and widely used test battery to assess motor skills, balance and coordination and to identify developmental delays and motor coordination difficulties in children and adolescents from 5 to 14 years (Aksay 2022). Previous studies have reported that the KTK test battery shows a test–retest reliability coefficient of 0.97 (Vandorpe et al. 2011), and it has been formerly employed in children and adolescents with cerebral damage and/or neurodevelopmental delay syndrome (Aksay 2022).

Specifically, the KTK3 battery was employed (Novak et al. 2017).

KTK3 maintains relatively high accuracy and has the potential to reduce the risk of injuries such as ankle sprains in children (*r* = 0.98, *p* < 0.001). The other three combined subtests require a total of 10 min, enhancing the practicality of the battery and ensuring full compliance from children.

The KTK3 procedure involves three trials: (1) walking backward (WB) on balance beams of three different widths (6, 4.5, and 3 cm), two times; (2) moving sideways (MS) using two boxes (25 × 25 × 5.7 cm) twice; (3) jumping sideways (JS) over a board (60 × 4 × 2 cm) as many times as possible within 15 s, repeated twice.

Trained supervisors conducted all assessments. All participants were given a demonstration and verbal instructions for the KTK3 battery.

### 2.5 Lower Body Strength

In the standing broad jump, the subjects stood behind the starting line, with feet together, pushed off vigorously and jumped forward as far as possible. The distance is measured from the take‐off line to the point where the back of the heel nearest to the take‐off line lands on the mat or non‐slippery floor. The test was repeated twice, and the best score was retained (in cm) (Castro‐Piñero et al. 2010). ICCs ranged between 0.89 and 0.98 (Tejero‐Gonzalez et al. 2013).

### 2.6 Cardiorespiratory Fitness

6MWT was conducted on flat, firm ground using a track of 30 m in length. Testing procedures adhered to the standardised protocols outlined by the American Thoracic Society (Enright 2003). Subjects were instructed to walk back and forth along a corridor marked by two orange plastic cones spaced 15–20 m apart. They were directed to walk as far as possible within 6 min without engaging in walking.

Participants were permitted to rest if needed but were instructed to resume walking as soon as they felt able. The total distance covered within the designated time frame was recorded to the nearest metre.

The 6MWT was supervised by either the treating physician or a trained sports science student to minimise variability. A familiarisation trial for the 6MWT was feasible before the start.

The ICC was fixed between 0.84 and 0.98 for Down syndrome subjects (Casey et al. 2012; Vis et al. 2009).

### 2.7 Cognitive Domain

The Cognitive Scale for Down Syndrome (CS‐DS, Startin et al. 2016) comprises 61 questions, offering three response options for informants to select from: ‘never/rarely true’, ‘sometimes true’, or ‘often/always true’. To mitigate response bias, half of the questions are reverse‐phrased. Scores range from 0 to 2 based on the response, wherein higher scores denote superior abilities. The CS‐DS questionnaire was filled in by participants' parents/authorised caregivers. Each evaluation was conducted in a private room with only the parent answering the questionnaire and the evaluator present to address any queries.

In our analysis, we utilised the total CS‐DS scores along with scores about memory, executive function and language domains (Startin et al. 2016). Test–retest reliability and interrater reliability were high; intraclass correlations were 0.95 (95% CI (0.91, 0.98), *p* < 0.001) for test–retest reliability and 0.84 (95% CI (0.74, 0.90), *p* < 0.001) for interrater reliability (Startin et al. 2016, 2019).

### 2.8 Statistical Analysis

Statistical analyses were conducted using IBM SPSS Statistics software, version [25]. A series of univariate analyses of variance (ANOVA) was performed to assess the impact of the protocol (pre: September vs. post: December) on various outcome measures.

The dependent variables included the KTK test (MS, JS, WB, KTK raw score), physical fitness (6MWT; SBJ) and a questionnaire for the executive function domain, memory domain and language domain.

Post hoc analyses, such as Tukey's honestly significant difference (HSD) tests, were conducted where appropriate to explore pairwise differences between the test months.

ANOVA was conducted to analyse the potential influence of protocol from September to December (pre vs. post).

Before conducting the ANOVA, assumptions including normality, homogeneity of variances and independence of observations were assessed. Where necessary, data transformations were applied to meet these assumptions. Additionally, the alpha level was set at 0.05 for all statistical tests.

## 3 Results

The clinical features of the participants before and at the end of the protocol are reported in Table 2. In 50% of children, obesity (BMI *z*‐score > 2) was detected.

**TABLE 2:** Clinical features of the participants before (T0) and at the end (T1) of the intervention programme.

	T0	T1	*p*
Age (years)	13.7 ± 2.34	—	—
Height (cm)	145 ± 9.68	144 ± 11.1	0.30
Weight (kg)	51.1 ± 11.9	45.1 ± 12.1	0.22
BMI (kg/m^2^)	24.4 ± 5.05	21.6 ± 4.74	0.36
BMI *z*‐score	1.53 ± 1.3	0.68 ± 1.08	0.91
Waist circumference (cm)	77.7 ± 12	71.7 ± 11	0.19
Waist circumference/height ratio	0.53 ± 0.09	0.49 ± 0.07	0.33
Systolic blood pressure	110 ± 10.1	97.9 ± 9.9	0.04
Diastolic blood pressure	66.1 ± 7.39	59.3 ± 3.45	0.20

A significant improvement in systolic BP was noted (*p* = 0.04). A decrease in adiposity parameters, including weight, BMI, BMI *z*‐score, WC and WC/H was also noted, without reaching statistical significance.

### 3.1 Gross Motor Coordination

**FIGURE 1:**
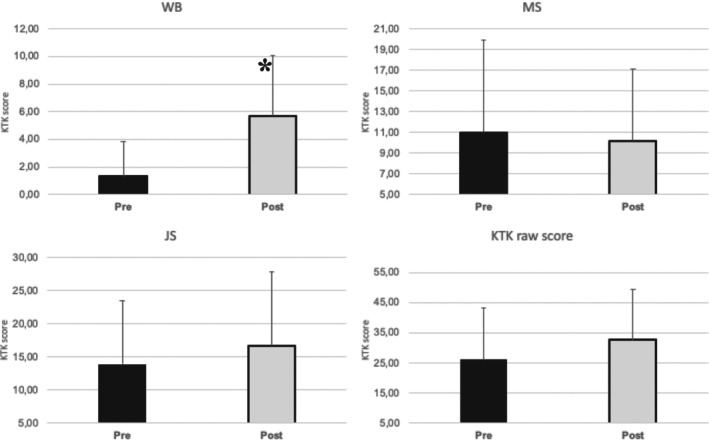
Comparison of KTK pre‐ and post‐intervention. *p < 0.05.

Comparison between items of KTK is in Figure 1. Analyses of balance through WB items showed a significant difference observed between pre‐ (1.41 ± 2.60) and post‐ (5.70 ± 4.37) intervention (*p* = 0.002).

Further, MS analysis revealed no significant association (pre: 11.65 ± 8.73; post: 10.20 ± 6.91; *p* = 0.808). Similarly, JS found no significant differences (pre: 14.00 ± 9.92; post: 16.70 ± 11.11; *p* = 0.490). The total KTK score, reflecting participants' gross motor coordination, did not vary significantly pre‐ and post‐intervention (pre: 27.06 ± 17.17; post: 32.60 ± 16.86; *p* = 0.349).

### 3.2 Physical Fitness

Physical fitness tests did not show differences from pre‐ to post‐evaluation. 6MWT analysis revealed no differences in cardiorespiratory fitness (pre: 367.38 ± 101.74; post: 421.12 ± 74.88; *p* = 0.119) and SBJ (pre: 81.69 ± 35.87; post: 90.12 ± 27.12; *p* = 0.609).

### 3.3 Child Developmental Disabilities Screening Questionnaire

No difference pre‐ and post‐intervention was noted in the sum of executive function (pre: 38.35 ± 4.91; post: 38.86 ± 4.74; *p* = 0.787) and memory (pre: 18.24 ± 3.24; post: 20.71 ± 1.38; *p* = 0.076) domain scores. Similarly, the analysis did not reveal any association in language domain scores (pre: 9.12 ± 2.00; post: 10.29 ± 2.29; *p* = 0.190).

## 4 Discussion

The present research is a pilot study that examined the effect of tele‐coaching training on adolescents with Down syndrome, focusing on motor coordination, physical fitness and cognitive domains. Improvements in balance have been observed, but there have been no differences in cognitive domains. A decrease in systolic BP was also detected.

Based on our knowledge, this is one of the first studies that involved subjects with Down syndrome and online exercise. The results of this pilot study provide initial insights into the effectiveness of an intervention for enhancing motor skills in adolescents with Down syndrome, particularly on balance assessed with WB item (KTK test). On one hand, in the applied practice context, even JS and overall KTK increased from pre‐ to post‐intervention. This suggests a potential benefit of the online exercise approach in specific areas of motor skill development. It has the potential to be a feasible intervention method. In fact, considering the adapted physical activity domain, even Wilroy et al. (2023) in a recent pilot study based on subjects with spinal cord injury highlighted the importance of innovative methods that aim to increase the adoption of sustainable and inclusive physical activity. However, the partial results obtained from this study can help in the future the creation of distance training programmes even more effective. Indeed, an increase in motor skills in Down syndrome could decrease sedentarism through Down syndrome with a potential increase in socialisation and quality of life (Muñoz‐Llerena et al. 2024; Post and Kraemer 2023). Furthermore, the lack of improvements in physical fitness outcomes (cardiorespiratory) was in contrast with some studies' findings highlighting the importance of assessing individual variability during the plan of training (Jacinto et al. 2023). Nevertheless, Dodd and Shields (2005) reported a possible overestimation of the effects reported by different cardiovascular training programmes for people with Down syndrome due to the lack of blind groups in many studies. This suggests that caution is needed in interpreting the extent of improvements observed. In fact, the relative VO2 peak was a crucial indicator of cardiovascular fitness. It is determined by cardiac blood flow, mainly affected by the volume of systolic ejection. Increases in ejection volume due to training are expected to lead to improvements in VO2 peak (Åstrand 2003). Indeed, another explanation for the lack of improvement in cardiorespiratory fitness in our sample could be that people with Down syndrome may have limited VO2 peak enhancement due to the physiological abnormalities of their condition of Down syndrome, such as heart problems, pulmonary hypoplasia and hypotonia (Cilhoroz et al. 2022). Reduced maximum heart rate during intense exercise associated with Down syndrome may further impair this ability, as VO2 peak depends on cardiac output (Cilhoroz et al. 2022; Seron and Greguol 2014).

In parallel with the increase in motor skills, another goal was to stimulate executive function through the game (Barnett et al. 2022). The absence of variation in executive function is not surprising, indeed Lanfranchi et al. (2010) suggest that adolescents with Down syndrome generally show an extensive impairment of executive functions. Further, our results are in line with other research based on subjects with Down syndrome, where attempts to train EF have not been easy and have had modest effects (Tungate and Conners 2021). However, emerging evidence suggests that long‐term physical activity and even mindfulness meditation can affect EF (Takacs and Kassai 2019). Diamond (2020) suggests effective interventions do not require long periods; instead, they reduce stress, increase social interaction, improve self‐confidence and are engaging. Therefore, physical activity and mindfulness might be the pathways for future EF interventions involving individuals with Down syndrome.

Many children with Down syndrome exhibit unfavourable cardiometabolic risk profiles, largely attributable to physical inactivity and unhealthy dietary habits. Additionally, to positive effects on motor skills, in our group, an improvement in BP was also noted. Recently, a significant inverse association between balance function and CVD‐related mortality risk was reported (Cao et al. 2021). Poor balance can result in a sedentary lifestyle (Yang et al. 2019) increasing the risk of CVD (Wilmot et al. 2012). As noted in the adult population, a balance improvement may be useful to maintain and ameliorate walking capacity and speed, inducing a cycle of increased physical activity that has a direct effect on cardiac health. This underscores the potential of exercise therapy to aid in restoring autonomic function and potentially mitigating the onset of cardiovascular comorbidities in individuals with Down syndrome (Calcaterra, Gazzarri, et al. 2023; Calcaterra, Schneider, et al. 2023; Dimopoulos et al. 2023; Sobey et al. 2015; Wang et al. 2023).

Lastly, increasing the duration of exercise sessions may prove beneficial in more clearly elucidating the effects of excess weight. However, based on our knowledge, this is the first study that uses online exercise in Down syndrome and the first study that analyses gross motor coordination through KTK3 in Down syndrome subjects.

The findings of this pilot study contribute to the growing body of evidence on the effectiveness of online exercise interventions for individuals with Down syndrome. Exploring the feasibility of incorporating these interventions into existing community support programmes for individuals with Down syndrome could enhance access to tailored interventions and support services. In accordance with the preliminary findings of this pilot study, future research could explore the long‐term effects of online exercise interventions on motor skill development and executive function in adolescents with Down syndrome. Investigating the optimal dosage, intensity and duration of these programmes, as well as identifying strategies to enhance participant engagement and adherence, can further optimise intervention. Comparative studies evaluating the effectiveness of online exercise interventions versus traditional in‐person interventions can provide valuable insights into their relative efficacy and cost‐effectiveness.

### 4.1 Limitations

Despite the promising results observed in certain areas, it is important to acknowledge the study's limitations. Our study represents a preliminary and pilot study. However, the small sample size and the absence of a control group may have influenced the findings. Additionally, the exclusion criteria may have limited the generalisability of the results to individuals with Down syndrome who have comorbidities or engage in specific habits. Finally, we acknowledge that IQ levels are not detailed in the present study. Even though cognitive performance could influence motor performance, excluding children with serious cognitive difficulties helps mitigate the risk of them not understanding the activities to be carried out.

## Author Contributions

Conceptualization: M.V. and V.C. Methodology: M.G., A.G. and V.C.P. Software: A.G. Validation: A.B. Formal analysis: M.G. and A.G. Investigation: A.P. Resources: V.C. and G.Z. Data curation: M.G. and A.G. Writing – original draft preparation: M.G., A.P., C.C., S.Z., A.Q. and A.A.G. Writing – review and editing: V.C.P, V.C., M.V. and G.Z. Visualisation: V.C. Supervision: M.V. Project administration: V.C. Funding acquisition: V.C. and M.V. All authors have read and agreed to the published version of the manuscript.

## Ethics Statement

The study protocol received approval from the Institutional Review Board (protocol number: 2020/ST/298; 2023/EM/099, CE Area 1 Milan, Italy).

## Consent

Upon receiving information regarding the study's nature, written informed consent was obtained from the parents/authorised caregivers of the participating children.

## Conflicts of Interest

The authors declare no conflicts of interest.

## Data Availability

The data that support the findings of this study are available from the corresponding author upon reasonable request.
